# Effects of Different Heterogeneous Nutrient Environments on the Growth and Activities of Enzymes in the Roots of *Fokienia hodginsii* Families

**DOI:** 10.3390/plants12244152

**Published:** 2023-12-13

**Authors:** Mi Deng, Bingjun Li, Yanmei Pan, Wenchen Chen, Tianyou He, Jundong Rong, Liguang Chen, Yushan Zheng

**Affiliations:** College of Forestry, Fujian Agriculture and Forestry University, Fuzhou 350002, China; sweetmi0609@126.com (M.D.); fafulbj@163.com (B.L.); panyanmei@163.com (Y.P.); cerdwin2003@163.com (W.C.); hetianyou1985@163.com (T.H.); rongjd@126.com (J.R.); clguang_cn@163.com (L.C.)

**Keywords:** *Fokienia hodginsii*, family, heterogeneous nutrients, grow, antioxidant enzyme activity

## Abstract

Currently, research on the *F. hodginsii* asexual lineage primarily focuses on the screening of growth traits and the control of single fertilizer applications. The effects of the heterogeneity of soil nutrients on root growth and activity have not been studied in detail. Therefore, we propose forest management measures to improve the foraging ability of forest trees in conjunction with stand productivity. In this experiment, annual containerized seedlings of 10 free-pollinated *F. hodginsii* lines from a primary asexual seed orchard were used as test subjects, and three heterogeneous nutrient environments of nitrogen (N), phosphorus (P), and potassium (K) were constructed. In contrast, homogeneous nutrient environments were used as the control to carry out potting experiments, to study the growth of *F. hodginsii* lines and the differences in the activities of root enzymes under the three heterogeneous nutrient environments, and to carry out the comprehensive evaluation using the principal component and cluster analysis method. The results were as follows: (1) The seedling height of *F. hodginsii* family lines under a homogeneous nutrient environment was significantly higher than that of all heterogeneous nutrient environments; the diameter of the ground was the highest under N heterogeneous nutrient environment and significantly higher than that of all the other nutrient environments; the biomass of the root system was the highest under P heterogeneous nutrient environment, which was significantly higher than that of homogeneous nutrient environment and K heterogeneous nutrient environment. The catalase (CAT) activity of *F. hodginsii* roots was higher than that of homogeneous nutrients in all heterogeneous nutrient environments but not significant, and the superoxide dismutase (SOD) activity was slightly higher than that of K heterogeneous and homogeneous nutrient environments in N and P heterogeneous nutrient environments. SOD activity was slightly higher than that of K heterogeneous and homogeneous nutrient environments under N, and P. peroxidase (POD) activity in the *F. hodginsii* root system was the highest under the P heterogeneous nutrient environment, which was significantly higher than that of the other nutrient environments. Unlike the activities of the enzymes, the content of malondialdehyde (MDA) in the roots of *F. hodginsii* was higher in the heterogeneous environment than in all the other nutrient environments. (2) Under N and P heterogeneous nutrient environments, lines 552 and 590 had higher seedling height, ground diameter, and root enzyme activity, while root biomass was highest in line 544; and under K heterogeneous nutrient environments, line 591 had higher seedling height, ground diameter, and root enzyme activity while root biomass was highest in line 551. In contrast to the patterns of seedling height, accumulation of root biomass and activities of root enzymes, family No. 590 had the highest ground diameter of all the *F. hodginsii* families under the heterogeneous nutrient environments. Family No. 547 had the highest MDA content. In conclusion, it can be seen that N heterogeneous and homogeneous nutrient environments can significantly increase the seedling height and diameter of *F. hodginsii* compared with P and K heterogeneous nutrient environments, and N and P heterogeneous nutrient environments can also increase the root biomass, root enzyme activities and significantly reduce the MDA content of *F. hodginsii*. According to the principal component analysis and cluster analysis, it can be seen that among the 10 *F. hodginsii* family lines, family lines 590 and 552 have higher evaluation in growth, root biomass accumulation, and enzyme activity.

## 1. Introduction

Soil is the most crucial carrier of plant nutrients. The distribution of soil nutrients in natural ecosystems is generally heterogeneous because different forest soils undergo leaching, immobilization, and soil biochemistry. Significant differences in texture, water content, microbial activity, and decomposition result in apparent gradients and patches of soil nutrients in spatial distribution [1,2,3]. Plants usually acquire more nutrient resources in heterogeneous nutrient environments compared with homogeneous ones [4,5]. Studies have shown that when the plant root system faces heterogeneous nutrient environments during stochastic growth, it produces different growth signals based on environmental heterogeneity. This then induces the root system to proliferate rapidly in nutrient-rich patches through synergistic effects, which then quickly leads the roots to occupy, excavate, and absorb effective resources in the patches. This process also induces the root system to undergo significant proliferation and expansion, which alters the strength of its physiological responses. In turn, this affects the growth and development of the plant [6,7]. Wang et al. [8] found that compared with the seeds obtained from Xinyi in Guangdong Province, China, those obtained from Cenxi, Guangxi, and Wuping in Fujian Province were more effective at colonizing and foraging for nutrients in heterogeneous nutrient environments compared with homogeneous nutrient environments. This is apparent in the differing abilities of the varying seed sources of *Pinus massoniana* to acquire heterogeneous nutrients. This observation suggests that there are enormous differences in the mechanism of foraging for nutrients and features of different plant genotypes, and they are adapted in a different way to heterogeneous nutrient environments and have varying degrees of sensitivity [9,10]. Drew et al. [11] found that a localized supply of NH_4_^+^–N, NO_3_^−^–N, or P all stimulated the proliferation of *Hordeum vulgare* roots, but localized K did not have such a stimulatory effect through the construction of potting experiments in heterogeneous nutrient environments. Brouder and Cassman et al. [12] studies on *Gossypium* root systems similarly confirmed that localized N and P applications had a stimulatory effect on root proliferation. In contrast, K did not have such a stimulatory effect. Rose et al. [13] found that the root systems of *Brassica napus* and *Triticum aestivum* responded more strongly to a localized supply of P than those of *Lupinus angustifolius*. Li et al. [14] found that *F. hodginsii* family lines’ photosynthetic intensity and fluorescence parameter levels increased in N and P heterogeneous nutrient environments compared with P and homogeneous nutrient environments. This suggested that the effects of the heterogeneous distribution of soil resources on stands are influenced by the characteristics of the patch and the type of nutrient; these differential effects are in addition to the differences in the genotypes of the stands themselves [15]. It was found that the spatial heterogeneity of soil-available nutrient resources on tilled and non-tilled soils was significant, ranging from 7 to 26 m in non-tilled soils and from 48 to 108 m in tilled soils. Tilled soils reduced the spatial heterogeneity of soil resources due to tillage disturbances, which resulted in a more homogeneous spatial distribution of soil resources. The minor scale of 7 m in non-tilled soils was only possible for trees or shrubs with large root ranges to respond to nutrient heterogeneity [16]. Hence, compared with crops and herbaceous plants, forest trees encounter more heterogeneous nutrient environments in their growth, perceive them on a larger scale, and acquire heterogeneous nutrient resources in a more genetically different manner between and within species [17,18]. Plant responses to spatial heterogeneity of soil nutrients and foraging mechanisms have become one of the hot spots of ecological research. Is there any difference in plant growth and root activity in heterogeneous nutrient environments? This is also a popular research topic in recent years [19].

*Fokienia hodginsii* [Dunn] Henry et Thomas is an evergreen tree in the Cupressaceae family. *F. hodginsii* is light-loving, shade-tolerant at seeding age, suitable for slightly acidic to acidic yellow and yellow-brown soils, shallow-rooted, drought and infertile, with developed lateral roots and no obvious main roots. These features encouraged its use as a pioneer species for afforestation in China [20]. Research on germplasm resources focuses on the distribution, population structure, exploitation value and genetic diversity of germplasm resources. Selection and breeding of superior germplasm resources is an essential part of the genetic improvement of *F. hodginsii*, and the abundant genetic variation can lay an excellent genetic foundation for Fujian cypress germplasm selection and improvement. Lineage selection refers to the process of selecting excellent lines and eliminating inferior lines according to the average value of the traits of the offspring from the free-pollinated offspring or controlled-pollinated offspring of the selected single plants [21]. However, research on the *F. hodginsii* asexual lineage primarily focuses on the screening of growth traits and the control of single fertilizer applications [22]. The effects of the heterogeneity of soil nutrients on root growth and activity have not been studied in detail. The assessment of the adaptability of different asexual lineages of forest trees to varying heterogeneous nutrient environments can help to generate an in-depth study on the foraging strategies and regulatory mechanisms of the belowground parts of trees in heterogeneous forests. Therefore, we propose forest management measures to improve the foraging ability of forest trees in conjunction with stand productivity [23,24]. Thus, this study selected 10 excellent *F. hodginsii* families as experimental materials, constructed three types of heterogeneous nutrient environments of N, P and K, and used the homogeneous nutrient environments as the control. We conducted potting experiments to study the growth of different *F. hodginsii* families and the differences in the activities of enzymes in the roots in variable nutrient environments. We then comprehensively evaluated these factors to provide a scientific and theoretical basis to cultivate foraging and highly efficient *F. hodginsii* families, as well as develop *F. hodginsii* plantations in a sustainable and high-yielding manner.

## 2. Results

### 2.1. Effects of Different Nutrient Environments on Growth and Root Biomass of F. hodginsii Families

The results of a two-way ANOVA (Table 1) showed that there was a highly significant interaction (*p* < 0.01) between family and nutrient environment on the seedling height and accumulation of root biomass of *F. hodginsii*, while there was no significant interaction (*p* > 0.05) between the two on their ground diameter. In terms of individual factors, the effects of the family line and nutrient environment on the seedling height, ground diameter and root biomass of *F. hodginsii* reached significant (*p* < 0.05) or highly significant (*p* < 0.01) levels.

**Figure 1:**
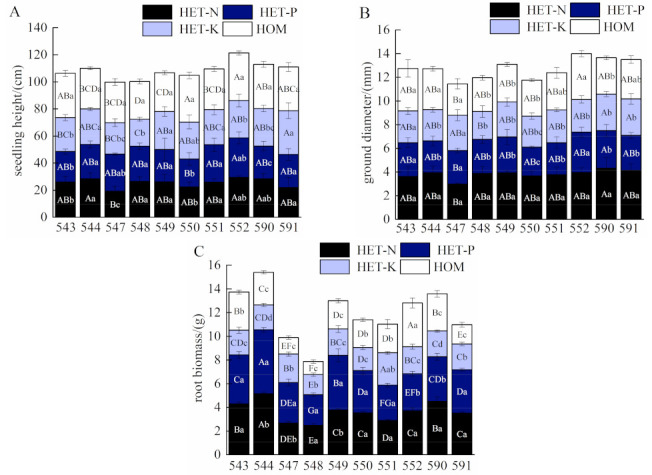
Effects of different nutrient environments on the growth and root biomass of *F. hodginsii* families. Note: Different lowercase letters represent significant differences between different nutrient environments in the same family (*p* < 0.05); different uppercase letters represent significant differences between different *F. hodginsii* families in the same nutrient environment (*p* < 0.05). (**A**) represents the comparison figure of seedling height; (**B**) represents the comparison figure of ground diameter; (**C**) represents the comparison figure of root biomass.

As shown in Figure 1, the *F. hodginsii* seedlings in the homogeneous nutrient environments were significantly higher than those in all the heterogeneous nutrient environments (*p* < 0.05). The mean value was 31.39 cm, and it increased by 19.2%, 23.7%, and 24.6% compared with that in the N, P, and K heterogeneous nutrient environments, respectively. The N heterogeneous nutrient environment produced the largest diameter of *F. hodginsii*, with a mean value of 3.81 mm, which was significantly higher than that in all the other nutrient environments (*p* < 0.05). The diameter increased by 18.4%, 31.4%, and 36.0% compared with the diameters in the homogeneous nutrient environment, respectively, and the N and P heterogeneous nutrient environments, respectively. The mean root biomass of *F. hodginsii* was 3.73 g in the P-heterotrophic nutrient environment, which was slightly higher than that in the N-heterotrophic nutrient environment. However, it was significantly higher by 55.0% and 71.1% in the homogeneous nutrient environment and the K-heterotrophic nutrient environment, respectively (*p* < 0.05).

The growth and root biomass of different *F. hodginsii* lines differed significantly even under the same nutrient environment (Figure 1). Family 552 seedlings grew the highest under the N and P heterogeneous nutrient environments, which increased by 53.0% and 41.2%, respectively, compared with the seedlings from families 547 and 550. These families were the lowest under the N and P heterogeneous nutrient environments, respectively. No. 591 had the highest *F. hodginsii* seedlings under the K heterogeneous nutrient environment with 32.33 cm (Figure 1A). The root biomass was the highest in No. 544 under both the N and P heterogeneous nutrient environments. The measurements of these root biomass indicated an increase of 108.6% and 107.8%, respectively, compared with that of No. 548, which had the lowest amount of root biomass. In contrast, family 551 had the highest root biomass under the K heterogeneous nutrient environment at 2.73 g. This was an increase of 61.2% compared with the lowest, which was observed in family 548 (Figure 1C). Unlike the patterns of seedling height and root biomass, the ground diameter of *F. hodginsii* was the highest in the No. 590 family in all the heterogeneous nutrient environments (Figure 1B).

### 2.2. Effects of Different Nutrient Environments on Root Enzyme Activities of F. hodginsii Families

The results of a two-way ANOVA (Table 2) showed that there was a highly significant (*p* < 0.01) interaction between family and nutrient environment on the activities of enzymes in the four root systems of *F. hodginsii*. In terms of individual factors, the effects of family on the enzyme activities of all four root systems of *F. hodginsii* were highly significant (*p* < 0.01), while the nutrient environment only had highly significant (*p* < 0.01) effects on the activity of POD and the content of MDA of the *F. hodginsii* roots. It had no significant (*p* > 0.05) effects on the activities of CAT and SOD.

**Figure 2:**
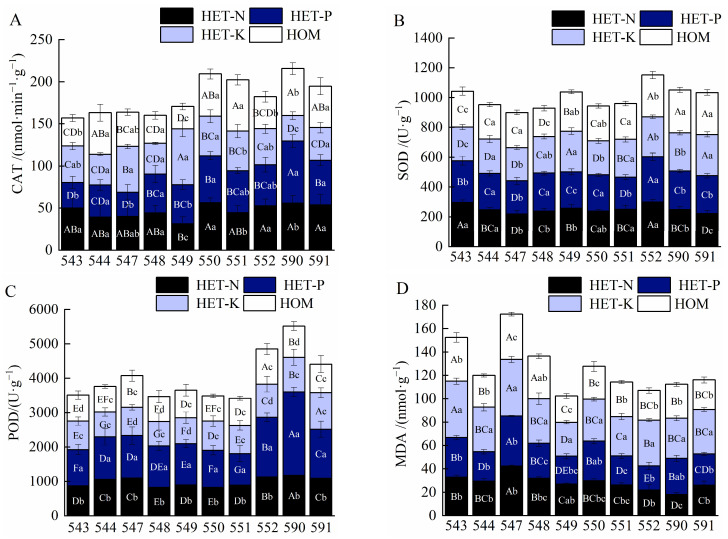
Effects of different nutrient environments on root enzyme activities of *F. hodginsii* families. Note: Different lowercase letters represent significant differences between different nutrient environments in the same family (*p* < 0.05); different uppercase letters represent significant differences between different *F. hodginsii* families in the same nutrient environment (*p* < 0.05). (**A**) represents the comparison figure of CAT; (**B**) represents the comparison figure of SOD; (**C**) represents the comparison figure of POD; (**D**) represents the comparison figure of MDA.

As shown in Figure 2A, the activity of CAT was higher in all the heterogeneous nutrient environments than in the homogeneous nutrient environments, but this difference was not significant (*p* > 0.05). Similar to the pattern of change in the activity of CAT, that of SOD was slightly higher in the N and P heterogeneous nutrient environments than in the K heterogeneous and homogeneous ones. However, there was no significant (*p* > 0.05) difference in the activity of SOD among all the nutrient environments (Figure 2B). *F. hodginsii* grown in the P heterogeneous nutrient environment had the highest activity of POD, with a mean value of 1355.33 U·g^−1^, which was significantly higher than that in all the other nutrient environments (*p* < 0.05). This value represented an increase of 37.8%, 59.0%, and 64.3% compared with that in the N, K heterogeneous and homogeneous nutrient environments, respectively (Figure 2C). *F. hodginsii* had the highest content of MDA and the lowest in the K and N heterogeneous nutrient environments, respectively. However, there was no significant difference in the content of MDA between the N and P heterogeneous and the homogeneous nutrient environments (*p* > 0.05, Figure 2C).

Under the same nutrient environment, the activities of enzymes in the roots and the contents of MDA in different *F. hodginsii* lines differed significantly. For example, the activities of CAT were the highest in lines 550, 590, and 549 under the three heterogeneous nutrient environments of N, P, and K, respectively, and increased by 80.3%, 156.4%, and 119.3%, respectively, compared with those of lines 549, 547, and 590. Lines 549, 547, and 590 had the lowest activities of CAT among the three nutrient environments (Figure 2A). Both the N and P heterotrophic nutrient environments in family 552 had the highest amount of activity of SOD, with increases of 36.5% and 39.9% in the former compared with the lowest in families 547 and 551, respectively. The highest levels of SOD activity were observed in family 591 in the K heterotrophic nutrient environments, with 275.89 U·g^−1^ (Figure 2B). POD was the most active in both the N and P heterotrophic nutrient environments in family 590, and both had their highest level of POD activity under both the N and P heterogeneous nutrient environments. The lowest level was observed in family No. 550, with the former increasing by 43.3% and 123.3%, respectively, compared with the latter. The POD in the K heterogeneous nutrient environments was the most active in family No. 591, with an increase of 50.0% compared with that of family No. 548, which had the lowest amount of activity (Figure 2C). Unlike the pattern of enzyme activities in the root system of *F. hodginsii*, the MDA content of *F. hodginsii* was highest in the No. 547 family and the lowest in the No. 552 family in all the heterogeneous nutrient environments (Figure 2D).

### 2.3. Correlation Analysis of Different Nutrient Environments on the Growth of F. hodginsii Families and Root Indexes

As shown in Table 3, the seedling height of *F. hodginsii* families was highly (*p* < 0.01) or significantly (*p* < 0.05) correlated with the diameter, SOD activity, root biomass and POD activity, and negatively (*p* < 0.05) correlated with the MDA content. The ground diameter of *F. hodginsii* was positively (*p* < 0.01) or significantly (*p* < 0.05) correlated with root biomass and SOD activity and negatively (*p* < 0.05) correlated with MDA content. The root biomass of *F. hodginsii* families was significantly (*p* < 0.05) positively correlated with POD activity. All three antioxidant enzyme activities were negatively correlated with MDA content, but only SOD activity reached the significant (*p* < 0.05) level.

### 2.4. Comprehensive Evaluation of Different Nutrient Environments on the Growth of F. hodginsii Families and Root Indexes

**Figure 3:**
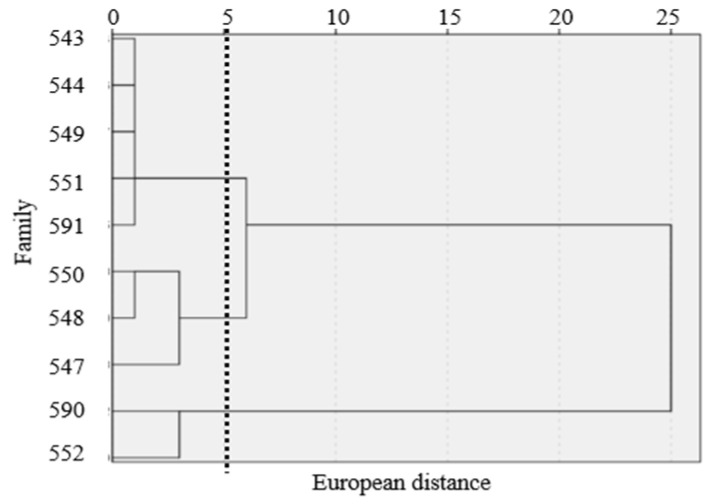
Cluster analysis of different nutrient environments on the growth and root indexes of *F. hodginsii* families.

A principal component analysis of seven growth and rooting indicators of the *F. hodginsii* family lines under different nutrient environments (Table 4) revealed that the cumulative rate of contribution of the first two principal components was 85.77%. This parameter basically reflected most of the information in the seven individual indicators. Thus, the first two principal components were adopted as the comprehensive evaluation indices. Combined with the eigenvectors, principal component 1 primarily included the seedling height, diameter and activity of SOD, which indicated that the higher eigenvalues of the variables described above significantly affected the growth and root development of the *F. hodginsii* lines. As shown in Table 4 and Table 5, the size of composite scores of the growth and root development of each *F. hodginsii* family under different nutrient environments were in the order of No. 552 > No. 590 > No. 544 > No. 591 > No. 549 > No. 543 > No. 551 > No. 550 > No. 548 > No. 547. Ward’s method was used to cluster and analyze the growth and root development of 10 *F. hodginsii* families, and a cluster dendrogram was established (Figure 3). Combined with the results of the composite scores from the principal component analysis, the seedling of the 10 *F. hodginsii* families were classified into three categories. Among them, numbers 552 and 590 were classified as Class I, with high evaluations on growth, accumulation of root biomass and enzyme activity. Numbers 544, 591, 549, 543, 551 and 550 were classified as Class II, with average evaluations on their growth, accumulation of root biomass and enzyme activity, and numbers 548 and 547 were classified as Class III, with low evaluations on these factors.

## 3. Discussion

### 3.1. Effects of Different Nutrient Environments on Growth and Root Biomass of F. hodginsii

The foraging behavior of forest trees is highly complex and varies not only by species or variety but also in relation to the nutrient environment of the soil [25]. The type of element and the nature of element distribution during plant growth affects the diameter, height and biomass of the aboveground portion of the forest trees [26,27]. In this study, we found that the height and diameter of the *F. hodginsii* seedlings were greater in the N heterogeneous and homogeneous nutrient environments than in the P and K heterogeneous ones. However, the root biomass was the highest in the P heterogeneous nutrient environments, and the root biomass in the P and N heterogeneous nutrient environments was significantly higher than those in the K heterogeneous and homogeneous ones. These findings indicated that the *F. hodginsii* seedlings that encountered the N, P and heterogeneous nutrient environments were more sensitive to growth, and they grew larger and had more root biomass. This indicates that the *F. hodginsii* seedlings that encountered the N and P-rich heterogeneous nutrient environments were more sensitive to growth and grew larger and had greater amounts of root biomass, while the *F. hodginsii* seedlings that encountered the K heterogeneous nutrient environments were not sensitive to growth. There was no obvious advantage in seedling height and the accumulation of dry matter when compared with the homogeneous nutrient environments. This indicated that the *F. hodginsii* seedlings differed substantially in their foraging behaviors when they encountered N, P, and K heterogeneous nutrient environments. This is similar to the findings of Ma et al. [28] and Jin et al. [29], which may be owing to the fact that P does not easily move in the soil. This results in an extremely stable P complex, which indicates that *F. hodginsii* has to proliferate its root system within the P heterogeneous nutrient environment if it requires the uptake of P from the soil. N moves more easily in the soil than P, and it is easily degraded and lost. Thus, the plant root system does not need to proliferate to obtain N. This results in a root biomass of *F. hodginsii* that is higher under a P heterogeneous nutrient environment than under an N heterogeneous nutrient environment [29]. The height of *F. hodginsii* seedlings and their diameter and root biomass in the K heterogeneous nutrient environment were lower than those in the other heterogeneous nutrient environments. This is probably owing to the fact that the content of K+ will inhibit the uptake of other nutrient elements in the *F. hodginsii* seedlings, which results in slow growth and inhibited accumulation of root biomass [30].

There are significant interspecific and intraspecific genetic differences in the nutrient-foraging mechanisms and characteristics of different plants. Yao et al. [25] found that the three seed sources of *Schima superba* were all highly adapted to their heterogeneous nutrient environments, and the accumulation of their dry matter and root length were significantly higher than those in the homogeneous nutrient environments. This study showed that the growth and root biomass of the *F. hodginsii* family lines differed significantly under the same nutrient environment. The seedlings grew the most in the No. 552 family line under the N and P heterogeneous nutrient environments, and the No. 591 family line grew the most under the K heterogeneous nutrient environment. The No. 544 family line had the highest amount of root biomass under the N and P heterogeneous nutrient environments, and the highest amount of root biomass in the No. 551 family line was observed under the K heterogeneous nutrient environment. In contrast to the changing pattern of the seedling height and root biomass, the diameter of the *F. hodginsii* family line was the highest in the No. 590 family line under all the heterogeneous nutrient environments, which could be the reason for the significant differences in the growth and root biomass of the *F. hodginsii* families. Unlike the pattern of seedling height and root biomass, the ground diameter of the *F. hodginsii* families was the highest in all the heterogeneous nutrient environments in the No. 590 family, which could be owing to the inconsistency of the degree of tolerance of *F. hodginsii* to nutrient environments with different elemental characteristics.

### 3.2. Effects of Different Nutrient Environments on Root Enzyme Activities of F. hodginsii

In addition to affecting the growth of *F. hodginsii*, the nutrient environment also regulates the activities of its root enzymes. The heterogeneous distribution of soil resources often causes changes in the activities of root enzymes in forest trees [31]. SOD, POD, and CAT mainly play a role in protecting the internal environmental mechanisms of plants against external stress and help plants to resist the damage of reactive oxygen species to plant cells, which can effectively improve the plant’s ability to adapt to and resist environmental stress. Their activities are often used as physiological and biochemical indicators of plant senescence [32]. In this study, we found that the activities of enzymes in the *F. hodginsii* roots under N and P heterogeneous nutrient environments were all slightly higher than those under the K heterogeneous and homogeneous nutrient environments. However, only the activity of POD reached the level of significance, which was somewhat similar to the findings of Zou et al. [33]. This may be because N and P factors are essential constituents of the vital activities of forest trees. The fine roots on one side of the plant, sensing the deprivation of N and P factors, can be induced to promote the growth of the root primordia and primary roots and increase the activity of root enzymes. With elevated metabolic levels, excess H_2_O_2_ is produced, which can cause damage to the plant organism. In order to avoid this damage, H_2_O_2_ must be quickly converted to other harmless or less toxic substances. POD has the dual role of eliminating H_2_O_2_ and phenolic and amine toxicity, so the POD activity of Fujian cypress roots under the environment of heterogeneous nutrients of N and P will be significantly increased to eliminate the harmful substances produced by the plant due to the metabolism of the elevated level of the plant, to obtain more N and P to meet the metabolic activities of physiology. This way, more N and P elements can be obtained to satisfy physiological metabolism [34]. When *F. hodginsii* encountered the K heterogeneous nutrient environment, there was less activity of the root enzymes. Its enzyme activity provided no obvious advantage in this environment compared with that of the homogeneous nutrient environment, which may be owing to the high content of K in the environment. *F. hodginsii* has low resistance to K, which can inhibit its own antioxidant enzymes. Thus, the seedlings in a K heterogeneous nutrient environment have less antioxidant enzyme activity than those that grow in N and P heterogeneous nutrient environments [35]. MDA is a peroxidation product of plant membrane lipids, and its content can reflect the degree of damage to the cell membrane system [36]. In this study, we found that the average content of MDA in *F. hodginsii* under the K heterogeneous nutrient environment was significantly higher than that of all the other nutrient environments. Among them, the lowest mean value of MDA content was found in the N heterogeneous nutrient environments. A more detailed analysis showed that the low resistance of *F. hodginsii* to K heterogeneous nutrient environments resulted in a greater degree of cell damage. Yu et al. [37] showed that the root system of *Cunninghamia lanceolata* could avoid damage by changing the activities of protective enzymes (SOD, CAT, and POD), inhibiting the formation of MDA, reducing the damage caused by membrane lipid peroxidation to the cell membrane system, and increasing the absorption of other nutrient elements to adapt to the heterogeneous low-P environment. This is consistent with this study. In addition, the activities of root enzymes and the contents of MDA in different *F. hodginsii* families varied significantly under the same nutrient environment. This indicated that different family lines of *F. hodginsii* have different adaptive abilities and sensitivities to the environment [38]. The same conclusion was also found in research on *Taxus wallichiana var. mairei* [39], *Schima superba* [40] and *Cunninghamia lanceolata* trees [41].

### 3.3. Effects of Different Nutrient Environments on the Growth and Root Enzyme Activities of F. hodginsii Families

Plants face a variety of environmental stresses during growth, which can lead to the generation and accumulation of free radicals in plant cells, which can impact plant growth and development. Antioxidant enzymes are a plant’s first line of defense against free radicals, and plants promote their growth and development by increasing the activity of protective enzymes or decreasing the level of lipid peroxidation [42]. In this study, we found that compared with K and homogeneous nutrient patches, the increase in enzyme activities and significant reduction in MDA content in *F. hodginsii* roots under N and P heterogeneous nutrient environments can defend the cell membrane from membrane lipid peroxidation and thus promote the growth of *F. hodginsii* seedlings, which may be attributed to the enhancement of antioxidant enzyme activities, the scavenging of free radicals, and the weakening of membrane lipid peroxidation in Fujian cypress seedlings under the N and P heterogeneous nutrient patch conditions. This may be because the N and P heterogeneous nutrient patch conditions stimulated the antioxidant enzyme activity of *F. hodginsii* seedlings, scavenging free radicals, weakening the membrane lipid peroxidation, thus reducing the MDA content, improving the plant’s ability to resist the environment, and promoting the growth of seedlings. In contrast, K and homogenous nutrient patches inhibit antioxidant enzyme activity, which is not conducive to the timely scavenging of reactive oxygen species, thus resulting in the accumulation of peroxidation products inhibiting plant growth and development. Ye et al. [43] found that compared to single NO_3_^−^–N, mixed NH_4_^+^–N, NO_3_^−^–N conditions in the rhizosphere of *Phyllostachys violascens* had low MDA content and antioxidant enzyme activities could be maintained at a high level, indicating that *Phyllostachys violascens* was subjected to a lower degree of membrane lipid peroxidation under the mixed nitrogen form of nutrient treatments, which resulted in a higher degree of resistance and, consequently, promoted the growth of the plant. Li et al. [44] found that N and P heterogeneous environments significantly increased root vigor and three kinds of antioxidant enzymes, reduced MDA content and promoted the growth of the height and diameter of *F. hodginsii* seedlings. At the same time, K heterogeneous environments had poorer effects on the growth and root development of *F. hodginsii* seedlings than homogeneous environments.

According to the results of the correlation analysis, SOD and POD were significantly positively correlated with root biomass, seedling height, and ground diameter, which indicated that the level of SOD and POD could reflect the accumulation and growth of plant root biomass. According to the results of principal component analysis, the absolute coefficients of seedling height, ground diameter, and SOD activity were significantly higher than other indexes, indicating that these physiological indexes were more sensitive to the growth environment of heterogeneous nutrient patches, and could better reflect the growth and root activity of *F. hodginsii* seedlings.

## 4. Materials and Methods

### 4.1. Overview of the Test Site

The experimental site was located in the greenhouse of the College of Horticulture, Fujian Agriculture and Forestry University, Fuzhou, China (119°13′51.18″ E, 26°05′4.35″ N). It is thoroughly ventilated and has spray cooling equipment and sunshade nets, among other amenities. During the experimental period, the average air temperature of the greenhouse ranged from 18 to 28 °C; the relative humidity was >78%, and the average duration of light was approximately 12 h. The sun shone from approximately 6:00 to 18:00.

### 4.2. Experimental Materials

Since 2019, this research group has carried out a multi-target breeding study on *F. hodginsii* in China and found abundant seed sources and family line variants, based on which 46 family lines were screened. Therefore, the test materials were 1-year-old container seedlings of 10 free-pollinated *F. hodginsii* families with excellent fruiting and growth traits from the primary asexual seed garden of *F. hodginsii* established in 2015 in the Fengtian State Forestry Farm, Anxi County, Quanzhou City, Fujian Province, with germplasm resource numbers of 543, 544, 547, 548, 549, 550, 551, 552, 590, and 591, respectively. The asexual lines of *F. hodginsii* that had been established in the seed garden originated from Fujian, Guangdong, Guangxi, Hunan, Guizhou, Zhejiang, Yunnan, Chongqing, Jiangxi and other provinces and regions. The asexual lines were from Fujian, Guangdong, Guangxi, Hunan, Guizhou, Zhejiang, Yunnan, Chongqing and Jiangxi provinces and regions. The seedlings were 18.30 ± 3.12 cm high on average, and their average diameter was 2.05 ± 0.53 mm. The potting substrate was an acidic, infertile red soil with a pH of 4.1, and the contents of hydrolyzed N, quick-acting K, and effective P were 35.7, 38.2, and 1.16 mg·kg^−1^, respectively.

### 4.3. Experimental Design

**Figure 4:**
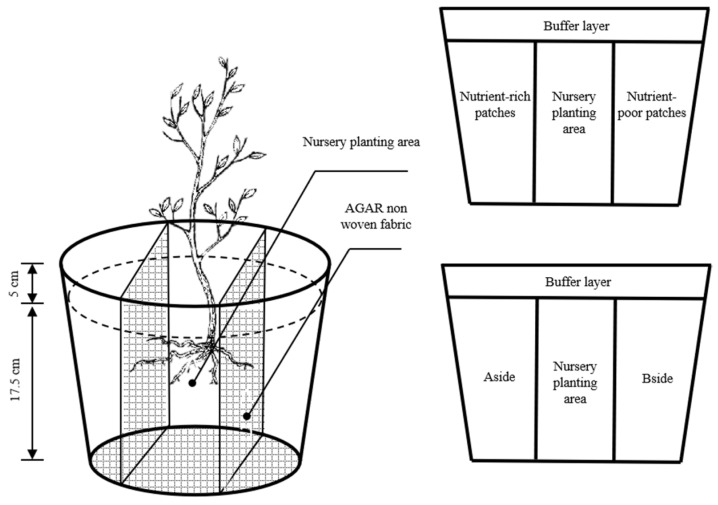
Schematic diagram of the device for constructing heterogeneous/homogeneous nutrient environments.

Polyethylene plastic pots that were 22.5 cm high with an inner diameter of 21 cm at the upper end and 15.5 cm at the lower end were selected as potting containers to construct the heterogeneous/homogeneous nutrient environments. The experiment began in early March 2022. The soil was first obtained from behind the Fujian Agriculture and Forestry University. Infertile, acidic red loam soil was brought back to the greenhouse for loosening. It was then disinfected with 0.5% potassium permanganate, covered with plastic film, sealed with exposure to air-drying for 1 week, and then passed through a sieve with a pore size of 5 mm and mixed well with perlite (mass ratio of 3:1) as the potting substrate. A total of 8 kg of substrate was added to each pot. As shown in Figure 4, the polyethylene plastic pots were divided into two parts. The upper part of the container was filled up to 5 cm with the substrate, and the lower part of the container was divided into three horizontal zones. From left to right, the pots contained the nutrient-rich patches/side A, the seedling planting area (the above potting substrate), and the nutrient-poor patch /side B. In this case, the two side regions were equal in volume and size and combined with each other to form heterogeneous (Nutrient-rich patches, nutrient-poor patches)/homogeneous (sides A and B) nutrient environment building zones. Each zone was separated by agar-coated non-woven fabric, which prevented the passage of nutrients but enabled the easy penetration of the primary and basal roots. Each polyethylene plastic pot had a layer of agar non-woven fabric at the bottom with a plastic tray to prevent nutrient loss.

As described by Mou et al. [45], equal amounts of N, P, and K were applied to both sides of A and B (0.1087 g of urea [46% N]), and 1.0081 g of calcium superphosphate (16% P_2_O_5_) and 0.1433 g of potassium chloride (60% K_2_O) were added per kg of substrate to construct a homogeneous nutrient environment. The two sides of A and B had concentrations of N, P, and K that were half of the total concentrations of the N, P, and K (50, 125, and 75 mg·kg^−1^, respectively). When the heterogeneous nutrient environment of the corresponding elements was constructed, no nutrients of the corresponding elements (0 mg·kg^−1^) were applied on the side of the nutrient-poor patch. The total concentration of the corresponding elements was applied on the side of the nutrient-rich patch. Thus, the nutrients of the two remaining elements were applied in both the nutrient-rich and nutrient-poor patches. The concentration of the nutrient that was applied was half of the total concentration of the nutrients of the remaining two nutrients. This concentration equaled the pattern of nutrients imposed under the homogeneous nutrient patches. The program used for the applications of the nutrients is shown in Table 6. The total concentrations of N, P and K applied in the heterogeneous nutrient environment were the same as those in the homogeneous nutrient environment. They were 100, 250 and 150 mg·kg^−1^, respectively.

There were four types of nutrient environments (N, P, and K heterogeneous nutrient environments and homogeneous nutrient environments), 10 *F. hodginsii* families, and 15 replicates for each treatment (i.e., 60 pots for each family). This resulted in a total of 600 pots, and one *F. hodginsii* seedling was planted in the center of each pot. The seedlings were transplanted at the beginning of March 2022, slowed down for 1 month, and the heterogeneous/homogeneous nutrient environment construction experiment was initiated on 10 April 2022. The plants were grown for 1 year, and distilled water was poured every 2 days after the beginning of the construction test. A volume of 300 mL of water was poured each time. The fertilizers were applied again in September 2022 and February 2023 at the same nutrient formulations and rates as the first application to fully ensure the survival of the heterogeneous/homogeneous nutrient environment in which the seedlings were placed. It should be noted that the seedlings have a long growth cycle.

### 4.4. Index Measurement

At the beginning of April 2023, eight seedlings of *F. hodginsii* with near-average growth of each family line under different treatments were selected to determine the height and diameter of the seedlings. The polyethylene plastic pots of the selected *F. hodginsii* seedlings were sliced open with scissors, and the entire *F. hodginsii* seedlings that were taken out were successively rinsed with tap water, followed by distilled water to wash the soil off the root system. The root surface was dried with absorbent paper, and the non-woven fabrics located at the two sides of the root system were then sliced open with scissors to retain the intact root tissues as much as possible. The roots were encapsulated with a self-sealing bag and placed in an ice box. They were immediately transported to the laboratory to be processed at low temperature. The root tissues of *F. hodginsii* from all the treatments were placed in a holding tank filled with ice for the test, and 15 root tip sites or white roots were selected from each treatment of each family line to determine the activities of antioxidant enzymes. The activities of superoxide dismutase (SOD), peroxidase (POD), and catalase (CAT) and the contents of malondialdehyde (MDA) in the root system were determined by assay kits from Suzhou Keming Biotechnology Co., Ltd. (Suzhou, China). Finally, the intact roots of each family line of *F. hodginsii* under different treatment conditions were placed in an oven, killed at 105 °C for 30 min, and then dried to a constant mass at 80 °C to determine the biomass of *F. hodginsii* roots.

### 4.5. Data Analysis

Microsoft Excel 2010 (Redmond, WA, USA) was used to enter the data and organize the tables. Nutrient environments and family lines were analyzed by using a two-way analysis of variance (ANOVA) using SPSS 23.0 (IBM, Inc., Armonk, NY, USA). A one-way ANOVA was used between different family lines of the same nutrient environment and between different nutrient environments of the same family line. Duncan’s method was used to compare multiple factors. The images were drawn using Origin 2018 (OriginLab, Northampton, MA, USA).

## 5. Conclusions

The N heterogeneous and homogeneous nutrient environments significantly increased the height and diameter of *F. hodginsii* seedlings in families compared with the families that were grown in the P and K heterogeneous nutrient environments. The N and P heterogeneous nutrient environments also increased the root biomass and activities of enzymes in the roots of the *F. hodginsii* families, as well as significantly reducing the content of MDA. The growth and root development of the *F. hodginsii* families were less sensitive to the K heterogeneous nutrient environment. In addition, the benefits of their growth in the K heterogeneous nutrient environment were not significantly different from those in the homogeneous nutrient environment. The principal component and cluster analyses of the growth and root system indices of different *F. hodginsii* families under varying heterogeneous nutrient environments revealed that families No. 552 and 590 had enhanced growth, accumulation of root biomass and enzyme activity. This study primarily focused on the aboveground and root physiological indices, and this research would benefit from a more thorough analysis. Therefore, in the next stage of this research, more root-related indices will be measured to further improve the experiment, with the aim of providing a theoretical basis for the seedling cultivation of *F. hodginsii* families and the selection of family lines. These data are important when selecting *F. hodginsii* families for silvicultural sites and understanding how to fertilize them appropriately.

## Figures and Tables

**Table 1:** Analysis of variance (ANOVA) of growth and root biomass of *F. hodginsii* families in different nutrient environments (*F* value).

Factor	Family	Nutrient Environment	Family × Nutrient Environment
seedling height	2.474 *	19.042 **	1.675 **
ground diameter	2.281 *	26.27 **	0.877 ^ns^
root biomass	104.721 **	551.43 **	25.391 **

*, *p* < 0.05; **, *p* < 0.01; ^ns^, *p* > 0.05.

**Table 2:** Analysis of variance of different nutrient environments on root enzyme activities of *F. hodginsii* families (*F* value).

Factor	Family	Nutrient Environment	Family × Nutrient Environment
CAT	6.246 **	1.57 ^ns^	5.92 **
SOD	112.57 **	1.926 ^ns^	11.16 **
POD	1232.28 **	6156.08 **	394.48 **
MDA	61.141 **	99.091 **	6.096 **

**, *p* < 0.01; ^ns^, *p* > 0.05.

**Table 3:** Correlation analysis of different nutrient environments on growth and root indexes of *F. hodginsii* families.

Parameter	Seedling Height	Ground Diameter	Root Biomass	CAT	SOD	POD	MDA
seedling height	1						
ground diameter	0.881 **	1					
root biomass	0.548 *	0.534 *	1				
CAT	0.392	0.257	0.032	1			
SOD	0.841 **	0.917 **	0.467	0.173	1		
POD	0.533 *	0.493	0.622 *	0.089	0.249	1	
MDA	−0.691 *	−0.700 *	−0.361	−0.490	−0.559 *	−0.288	1

*, *p* < 0.05; **, *p* < 0.01.

**Table 4:** Principal component analysis of growth and root indexes of *F. hodginsii* families in different nutrient environments.

Index	Principal Component	
	1	2
seedling height	0.948	−0.060
ground diameter	0.944	−0.004
root biomass	0.676	0.528
POD	0.602	0.525
SOD	0.851	−0.047
CAT	0.394	−0.716
MDA	−0.777	0.374
Eigenvalue	4.091	1.212
Contribution rate/%	58.45	27.32
Cumulative contribution rate/%	58.45	85.77

**Table 5:** Comprehensive evaluation results of different nutrient environments on the growth and root indexes of *F. hodginsii* families.

Family	543	544	547	548	549	550	551	552	590	591
Comprehensive scores	−0.11	0.91	−1.75	−1.62	0.32	−0.92	−0.43	1.84	1.30	0.45
Comprehensive rank	6	3	10	9	5	8	7	1	2	4

**Table 6:** N, P and K concentrations applied in constructing heterogeneous/homogeneous nutrient environments.

Nutrient Patch	Heterogeneous Nutrient Patch						Homogeneous Nutrient Patches					
	Nutrient-Rich Patches			Nutrient-Poor Patches			Side A			Side B		
	N	P	K	N	P	K	N	P	K	N	P	K
HET-N	100	125	75	0	125	75	50	125	75	50	125	75
HET-P	50	250	75	50	0	75						
HET-K	50	125	150	50	125	0						

## Data Availability

Data recorded in the current study are available in all Tables and Figures of the manuscript.

## References

[1] Hodge A. (2004). The plastic plant: Root responses to heterogeneous supplies of nutrients. New Phytol..

[2] Hodge A. (2006). Plastic plants and patchy soils. J. Exp. Bot..

[3] Wang X., Tang H.L., Shen J.B. (2013). Root responses of maize to spatial heterogenous nitrogen and phosphorus. J. Plant Nutr. Fertil..

[4] Yao J., Zhou Z., Chu X., Xu H., Tong J. (2018). Effect of neighborhood competition on dry matter accumulation, nitrogen and phosphorus efficiency of three provenances of *Schima superba* in a heterogeneous nutrient environment. Acta Ecol. Sin..

[5] Zhang Y., Zhou Z., Yang Q. (2013). Genetic variations in root morphology and phosphorus efficiency of *Pinus massoniana* under heterogeneous and homogeneous low phosphorus conditions. Plant Soil.

[6] Sun J.L., Li H.B., Zhang A.P. (2022). Effects of nutrient heterogeneity on shoot and root growth of *Zea mays* and intraspecific competition. J. China Agric. Univ..

[7] Grime J.P. (2007). The Scale-precision trade-off in spacial resource foraging by plants: Restoring perspective. Ann. Bot..

[8] Wang J., Zhou Z.C., Jin G.Q., Rao L.B., Jiao Y.L., Li Y.G. (2007). Differences of foraging behavior between provenances of *Pinus massoniana* in heterogeneous nutrient environment. Acta Ecol. Sin..

[9] Kyle C.K., Peairs S.E., Ezell A.W., Belli K.L., Hodges J.D. (2011). Understory Light Conditions Associated with Partial Overstory Removal and Midstory/Understory Control Applications in a Bottomland Hardwood Forest. Forests.

[10] Yan X.L., Ma X. (2021). Responses of root morphology and seedling growth in three tree species to heterogeneous supplies of ammonium and nitrate. For. Ecol. Manag..

[11] Drew M.C. (1975). Comparison of the effects of a localized supply of phosphate, nitrate, ammonium and potassium on the growth of the seminal root system, and the shoot in Barley. New Phytol..

[12] Brouder S.M., Cassman K.G. (1994). Evaluation of a Mechanistic Model of Potassium Uptake by Cotton in Vermiculitic Soil. Soil Sci. Soc. Am. J..

[13] Rose T.J., Rengel Z., Ma Q., Bowden J.W. (2009). Crop species differ in root plasticity response to localised P supply. J. Plant Nutr. Soil Sci..

[14] Li B., Deng M., Pan Y., Rong J., He T., Chen L., Zheng Y. (2023). Responses of Planting Modes to Photosynthetic Characteristics and Fluorescence Parameters of *Fokienia hodginsii* Seedlings in a Heterogeneous Nutrient Environment. Forests.

[15] Wang P., Mou P., Li Y. (2012). Review of root nutrient foraging plasticity and root competition of plants. Chin. J. Plant Ecol..

[16] Freschet G.T., Swart E.M., Cornelissen J.H.C. (2015). Integrated plant phenotypic responses to contrasting above- and below-ground resources: Key roles of specific leaf area and root mass fraction. New Phytol..

[17] Glover G.R., Zutter B.R. (1993). Loblolly pine and mixed hardwood stand dynamics for 27 years following chemical, mechanical, and manual site preparation. Can. J. For. Res..

[18] Grossman J.D., Rice K.J. (2013). Evolution of root plasticity responses to variation in soil nutrient distribution and concentration. Evol. Appl..

[19] Song P., Zhang R., Zhou Z., Tong J.S., Wang H. (2017). Effects of localized nitrogen supply treatments on growth and root parameters in *Pinus massoniana* families under phosphorus deficiency. Chin. J. Plant Ecol..

[20] Dao T.H.H., Hölscher D. (2017). Fujian cypress and two other threatened tree species in three conservation zones of a nature reserve in north-western Vietnam. For. Ecosyst..

[21] Tang D. (2023). Family Variation and Evaluation of Growth Traits On 36-year-old *Pinus massoniana* Lamb in Fujian Province. For. Res..

[22] Li B., Chen Q., Wang X.X., Rong J.D., Chen L.G., Zhen Y.S. (2022). Differences in Growth and Nutrients between Pure and Mixed Forest of *Fokienia hodginsii* with Different Forest Ages. Acta Bot. Boreali Occident. Sin..

[23] Zhen R.H., Yang Z.W., Shi J.S., Huang D.L., Huang X.M. (2003). Studies on the Growth rhythm and Genetic variations of traits among plus-tree progeny families of *Fokienia Hodginsii* at seedling stage. Sci. Silvae Sin..

[24] Wu P., Ma X., Tigabu M., Wang C., Odén P.C. (2011). Root morphological plasticity and biomass production of two Chinese fir clones with high phosphorus efficiency under low phosphorus stress. Can. J. For. Res..

[25] Yao J.B., Chu X.L., Zhou Z.C., Xu H.B., Zhen X.J. (2018). Response of Seedlings of Three *Schima superba* Provenances to Different Light Environments When Mixed Planting with *Cunninghamia lanceolata*. For. Res..

[26] Yan X.L., Hu W.J., Ma Y.F., Huo Y.F., Wang T., Ma X.Q. (2020). Nitrogen Uptake Preference of *Cunninghamia lanceolata*, *Pinus massoniana*, and *Schima superba* under Heterogeneous Nitrogen Supply Environment and their Root Foraging Strategies. Sci. Silvae Sin..

[27] Mommer L., Visser E.J.W., Ruijven J.V., Caluwe H.D., Pierik R., Kroon H.D. (2011). Contrasting root behaviour in two grass species: A test of functionality in dynamic heterogeneous conditions. Plant Soil.

[28] Ma X.H., Zhou Z.C., Zhang Y., Jin G.Q. (2010). Foraging Behaviors and Growth Responses of *Pinus massoniana* Seeding in the Heterogeneous Nutrient Environment with Different Nutrient Patches. For. Res..

[29] Jin S.H., Huang J.Q., Li X.Q., Zheng B.S., Wu J.S., Wang Z.J., Liu G.H., Chen M. (2011). Effects of potassium supply on limitations of photosynthesis by mesophyll diffusion conductance in *Carya cathayensis*. Tree Physiol..

[30] Wu P., Wang G., Farooq T.H., Li Q., Zou X., Ma X. (2017). Low phosphorus and competition affect Chinese fir cutting growth and root organic acid content: Does neighboring root activity aggravate P nutrient deficiency?. J. Soils Sediments.

[31] Sun B., Liao H., Su Y.H., Xu W.F., Jiang Y.J. (2015). Progress in the study of some key synergistic mechanisms affecting nitrogen and phosphorus utilization in soil-root-microbial systems. Soils.

[32] Li X., Zhang L., Li Y., Ma L., Bu N., Ma C. (2012). Changes in photosynthesis, antioxidant enzymes and lipid peroxidation in soybean seedlings exposed to UV-B radiation and/or Cd. Plant Soil.

[33] Zou X.H., Wu P.F., Jia Y.Y., Ma X.Q. (2016). Periodical response of Chinese fir root to the phosphorus concentrations in patches and heterogeneous distribution in different growing stages. J. Plant Nutr. Fertil..

[34] Yan M., Wu Y.M., Huang S.X., Huang X.L. (2011). Resistance Physiological Response of Different Fast-Growing Eucalyptus Clones to Acid-Aluminum Stresses. Sci. Silvae Sin..

[35] Chen B.J.W., During H.J., Vermeulen P.J., De Kroon H., Poorter H., Anten N.P.R. (2015). Corrections for rooting volume and plant size reveal negative effects of neighbour presence on root allocation in pea. Funct. Ecol..

[36] Wu R.J., Zhuang J., Huang J., Chen W.P. (2009). Responses and Resistance Mechanismof *Pinus massoniana* under the Stresses of Simulated Acid Rainand Aluminum. Sci. Silvae Sin..

[37] Yu D.J., Xia L.D., Yin D.Y., Zhou C.F. (2018). Effects of Phosphorus on Aluminum Tolerance of Chinese Fir Seedlings. Sci. Silvae Sin..

[38] Song P., Zhang R., Zhang Y., Zhou Z.C., Feng Z.P. (2016). Effects of simulated nitrogen deposition on fine root morphology, nitrogen and phosphorus efficiency of *Pinus massoniana* clone under phosphorus deficiency. Chin. J. Plant Ecol..

[39] Xiao Y., Chu X.L., Yin Z.F., Jiang J.M., Wang H., Zhou Z.C. (2016). Analyses on differences in seedling growth, photosynthetic physiology and height growth rhythm of each family of *Taxus wallichiana* var. mairei from different locations. J. Plant Resour. Environ..

[40] Yao J.B., Chu X.L., Zhou Z.C., Tong J.S., Wang H., Yu J.Z. (2017). Different responses of growth and root development of *Schima superba* provenance to the adjacent plant competition in different nutrient conditions. Chin. J. Appl. Ecol..

[41] Zhang J.J., Xu S.S., Cao G.Q., Lin S.Z., Pan Y.M., Ye Y.Q. (2020). Effects of Nitrogen Forms on the Chlorophyll Fluorescence Parameters and Chloroplast Ultra-structure of *Cunninghamia lanceolata*. J. Northwest For. Univ..

[42] Savicka M., Škute N. (2010). Effects of high temperature on malondialdehyde content, superoxide production and growth changes in wheat seedlings (*Triticum aestivum* L.). Ekologija.

[43] Ye L.S., Chen S.L. (2017). Antioxidant system response to different forms and ratios of nitrogen in leaves and roots of *Phyllostachys violascens*. J. Zhejiang A F Univ..

[44] Li B., Deng M., Pan Y., Chen W., Rong J., He T., Chen L., Zheng Y. (2023). Responses of Growth andRoot Vitality of *Fokienia hodginsii* Seedling to the Neighbor Competition in Different Heterogeneous Nutrient Environments. Forests.

[45] Mou P., Jones R.H., Tan Z., Bao Z., Chen H. (2013). Morphological and physiological plasticity of plant roots when nutrients are both spatially and temporally heterogeneous. Plant Soil.

